# Real‐world analysis of adverse event rates after initiation of ibrutinib among Medicare beneficiaries with chronic lymphocytic leukemia

**DOI:** 10.1002/cam4.6953

**Published:** 2024-02-01

**Authors:** Scott F. Huntington, Enrico de Nigris, Justin T. Puckett, Sachin Kamal‐Bahl, Mohammed Farooqui, Katherine Ryland, Eric M. Sarpong, Siyang Leng, Xiaoqin Yang, Jalpa A. Doshi

**Affiliations:** ^1^ Department of Internal Medicine, Section of Hematology Yale University School of Medicine New Haven Connecticut USA; ^2^ MSD (UK) Limited London UK; ^3^ COVIA Health Solutions Ambler Pennsylvania USA; ^4^ Merck & Co., Inc Rahway New Jersey USA; ^5^ Division of General Internal Medicine, Perelman School of Medicine University of Pennsylvania Philadelphia Pennsylvania USA

**Keywords:** adverse event, chronic lymphocytic leukemia, discontinuation, elderly, ibrutinib, Medicare

## Abstract

**Background:**

The first‐generation BTK inhibitor ibrutinib is a standard‐of‐care therapy in the treatment of chronic lymphocytic leukemia (CLL) despite potential side effects that often lead to discontinuation.

**Methods:**

This study used 2013–2019 claims data to describe the incidence rate of adverse events (AEs) among elderly Medicare beneficiaries newly initiating ibrutinib for CLL.

**Results:**

The final sample contained 11,870 Medicare beneficiaries with CLL (mean age 77.2) newly initiating ibrutinib, of whom 65.2% discontinued over mean follow‐up of 2.3 years. The overall incidence rate of AEs was 62.5 per 1000 patient‐months for all discontinuers and 32.9 per 1000 patient‐months for non‐discontinuers. Discontinuers had a higher incidence rate of AEs per 1000 patient‐months compared with non‐discontinuers for all AEs examined, including infection (22.8 vs. 14.5), atrial fibrillation (15.1 vs. 7.0), anemia (21.9 vs. 14.5), and arthralgia/myalgia (19.5 vs. 13.6).

**Conclusion:**

In this first real‐world study of a national sample of elderly US patients treated with ibrutinib, we found a clear unmet need for improved management of ibrutinib‐related AEs and/or new treatments to improve real‐world outcomes in patients with CLL.

## Graphical abstract

In a real‐world analysis of over 11,000 Medicare beneficiaries with CLL who initiated ibrutinib, the overall incidence rate of adverse events was 62.5 per 1000 patient‐months for all discontinuers and 32.9 per 1000 patient‐months for non‐discontinuers. Discontinuers had a higher incidence rate of AEs per 1000 patient‐months compared with non‐discontinuers for all AEs examined, largely driven by patients who discontinued within 12 months of ibrutinib initiation. These findings highlight a clear unmet need for improved management of ibrutinib‐related AEs and/or new treatments to improve real‐world outcomes in patients with CLL.

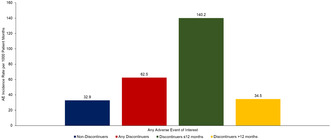

Chronic lymphocytic leukemia/small lymphocytic lymphoma (CLL/SLL), a malignancy of auto‐reactive mature B cells, primarily impacts the elderly and increases in prevalence with age.^1^ The first Bruton's tyrosine kinase inhibitor (BTKi) ibrutinib, a once‐daily oral medication, became a standard of care in the treatment of relapsed/refractory CLL/SLL soon after its US FDA approval in 2014 given significant improvements in progression‐free and overall survival relative to traditional chemotherapies.^2^, ^3^, ^4^ However, clinical studies have shown several adverse events (AEs) to be associated with ibrutinib use, some of which can lead to treatment discontinuation. Ibrutinib‐related AEs are a more common reason for its discontinuation in the real‐world setting than in clinical trials.^2^, ^5^, ^6^, ^7^ The majority of real‐world studies in the United States to date have been limited to patients treated at select academic centers (often with greater experience using ibrutinib) or focused on the commercially insured/younger population.^5^, ^7^, ^8^, ^9^ Hence, findings from these studies may not be representative of the AEs observed among elderly patients, who are more likely to be frail and have multiple comorbidities. The objective of this real‐world study is to describe the incidence rate of AEs observed after ibrutinib treatment initiation in a national sample of US Medicare beneficiaries with CLL/SLL, stratified by ibrutinib discontinuation status and timing of discontinuation.

This retrospective cohort study used 2013–2019 Medicare claims data (100%), the most recently available data at the time of the analysis. The Medicare data include comprehensive claims for all fee‐for‐service Medicare patients, including Medicare Parts A and B‐covered medical claims for inpatient care, skilled nursing facility care, home health services, outpatient services including physician‐administered drugs such as infusions, durable medical equipment, and hospice services as well as Medicare Part D prescription drug events (PDE) files for self‐administered outpatient drugs. All elderly fee‐for‐service Medicare beneficiaries who newly initiated ibrutinib between 01/01/2014 and 12/31/2018 (index date = first ibrutinib prescription claim date) were identified. Patients were required to meet the following criteria: (1) continuous Medicare Part A, B, and D coverage in 12 months before and at least 4‐months after the index date, (2) presence of a diagnosis of CLL/SLL in the 12‐month pre‐index period and at any time over follow‐up, (3) ≥66 years on index date, (4) absence of ≥2 diagnoses for another FDA‐approved indication of ibrutinib, and (5) absence of ibrutinib prescription in the 12‐month pre‐index period.

Patients were classified as discontinuers (defined as the presence of a consecutive 60‐day gap in ibrutinib treatment^9^, ^10^) or non‐discontinuers. Discontinuers were further stratified by whether they discontinued within 12 months of ibrutinib initiation or not. A selected list of hematologic and non‐hematologic AEs (see Table S1) was identified from the medical claims by the presence of an International Classification of Diseases, Ninth and Tenth Revision (ICD‐9/ICD‐10) code and were measured over the duration of ibrutinib treatment (i.e., from ibrutinib initiation date to date of ibrutinib discontinuation or in the case of non‐discontinuers until the end of the observation period). To ensure new cases of each of the AES were identified and reported, only patients *without* evidence of a specific AE in the 12‐month pre‐index period (i.e., prior to ibrutinib initiation) were reported as having the AE. The outcome of interest in this study was the AE incidence rate per 1000 patient‐months (i.e., [number of new AE cases / total person‐months] × 1000) reported by discontinuation status. The AE incidence rate per 1000 patient months was reported overall (i.e., for any AE of interest) and by type of individual AE of interest.

The final sample comprised of 11,870 Medicare patients who newly initiated ibrutinib treatment between January 1, 2014 and December 31, 2018 (see Table 2). The mean (SD) age was 77.2 (6.8) years; the sample was 58.5% male and 90.1% White (Table S3). Though non‐discontinuers and discontinuers were largely similar with respect to most characteristics, non‐discontinuers were slightly younger (mean age 76.5 years; 44.5% aged <75 years) than discontinuers (mean age 77.6 years; 37.2% aged <75 years) and had fewer comorbidities in the pre‐index period (i.e., atrial fibrillation 13.9% vs. 19.2%) (Table S3). Approximately two thirds (65.2%) of patients had evidence of being discontinuers whereas the remainder were non‐discontinuers over a mean follow‐up of 2.3 years. When stratified by timing of discontinuation, 45.1% of the overall sample had discontinued within 12 months, 20.1% had discontinued >12 months of ibrutinib treatment, and the remaining 34.8% were non‐discontinuers over the same follow‐up period.

The overall AE incidence rate per 1000 patient months is presented in Figure 1. The total number of patient‐months of observation from ibrutinib initiation date were 107,967 for non‐discontinuers, 85,132 for all discontinuers, 22,551 for discontinuers ≤12 months and 62,581 for discontinuers >12 months. The overall incidence rate including all examined AEs was 62.5 per 1000 patient‐months for all discontinuers and 32.9 per 1000 patient‐months for non‐discontinuers. The incidence rate in the subgroup of discontinuers ≤12 months was even higher relative to both non‐discontinuers (140.2 vs. 32.9 AEs per 1000 patient months) and discontinuers >12 months (140.2 vs. 34.5 AEs per 1000 patient months).

**FIGURE 1:**
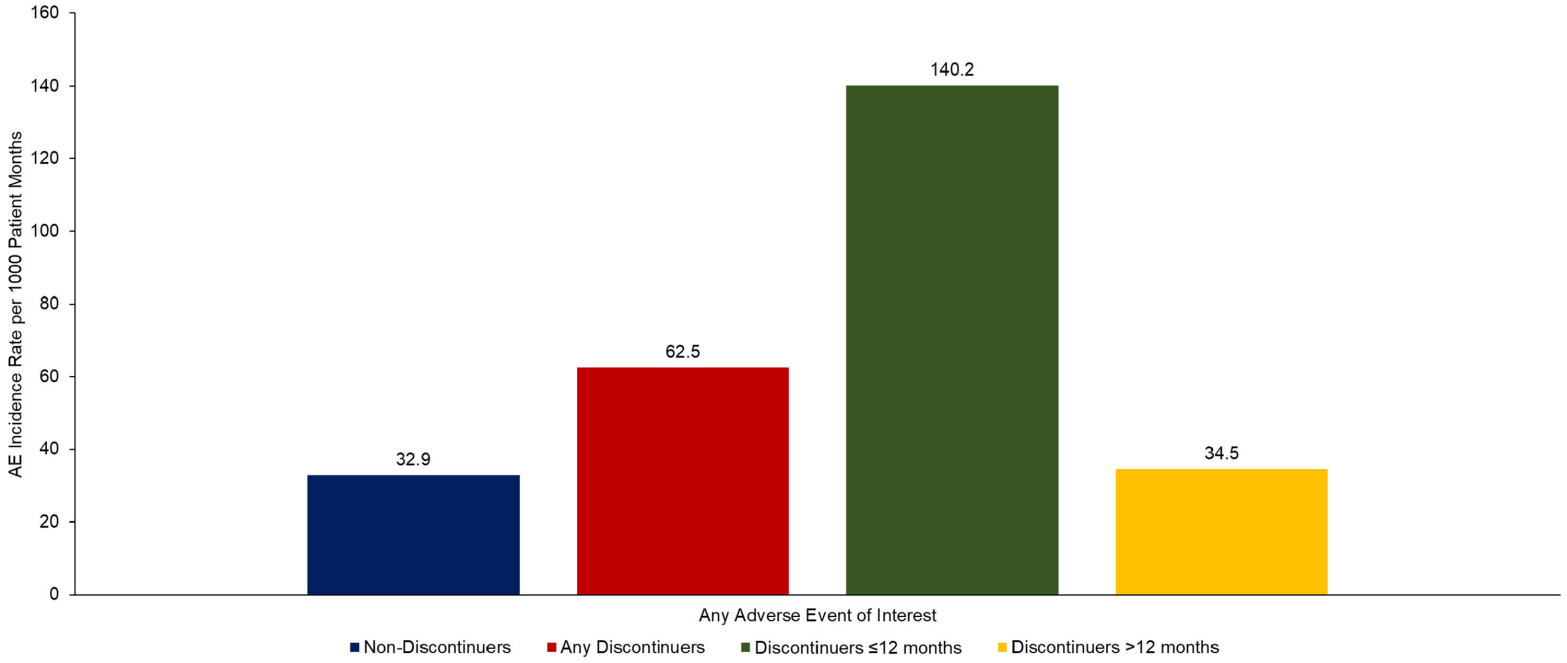
Overall incidence of any* adverse event per 1000 patient‐months of ibrutinib treatment among elderly Medicare beneficiaries with CLL/SLL initiating ibrutinib by discontinuation status and timing of discontinuation. The total number of person‐months on ibrutinib treatment for non‐discontinuers (i.e., total patient‐months from ibrutinib initiation date until end of follow‐up) were 107,967. Total number of person‐months on ibrutinib treatment for discontinuers (i.e., total patient‐months from ibrutinib initiation date until its discontinuation) were 85,132 for any discontinuers, 22,551 for discontinuers ≤12 months and 62,581 for discontinuers >12 months. *Includes any adverse event of interest listed in Table S1.

The AE incidence rate per 1000 patient months by type of AE is presented in Figure 2 and Table S4. The most common AEs observed in both groups of discontinuers and non‐discontinuers were hematologic AEs of anemia and thrombocytopenia and non‐hematologic AEs of infections, arthralgia/myalgia, and cardiovascular comorbidities such as atrial fibrillation, heart failure, and ventricular arrhythmia. However, discontinuers had a higher incidence rate of AEs compared to non‐discontinuers for each of the AEs examined. For instance, the incidence rate of atrial fibrillation per 1000 patient‐months was 7.0 in non‐discontinuers, 15.1 among all discontinuers, 30.2 among discontinuers ≤12 months, and 9.7 among discontinuers >12 months; similarly, incidence rate of any bleeding was 4.9, 11.4, 25.6, and 6.3 per 1000 patient‐months, respectively.

**FIGURE 2:**
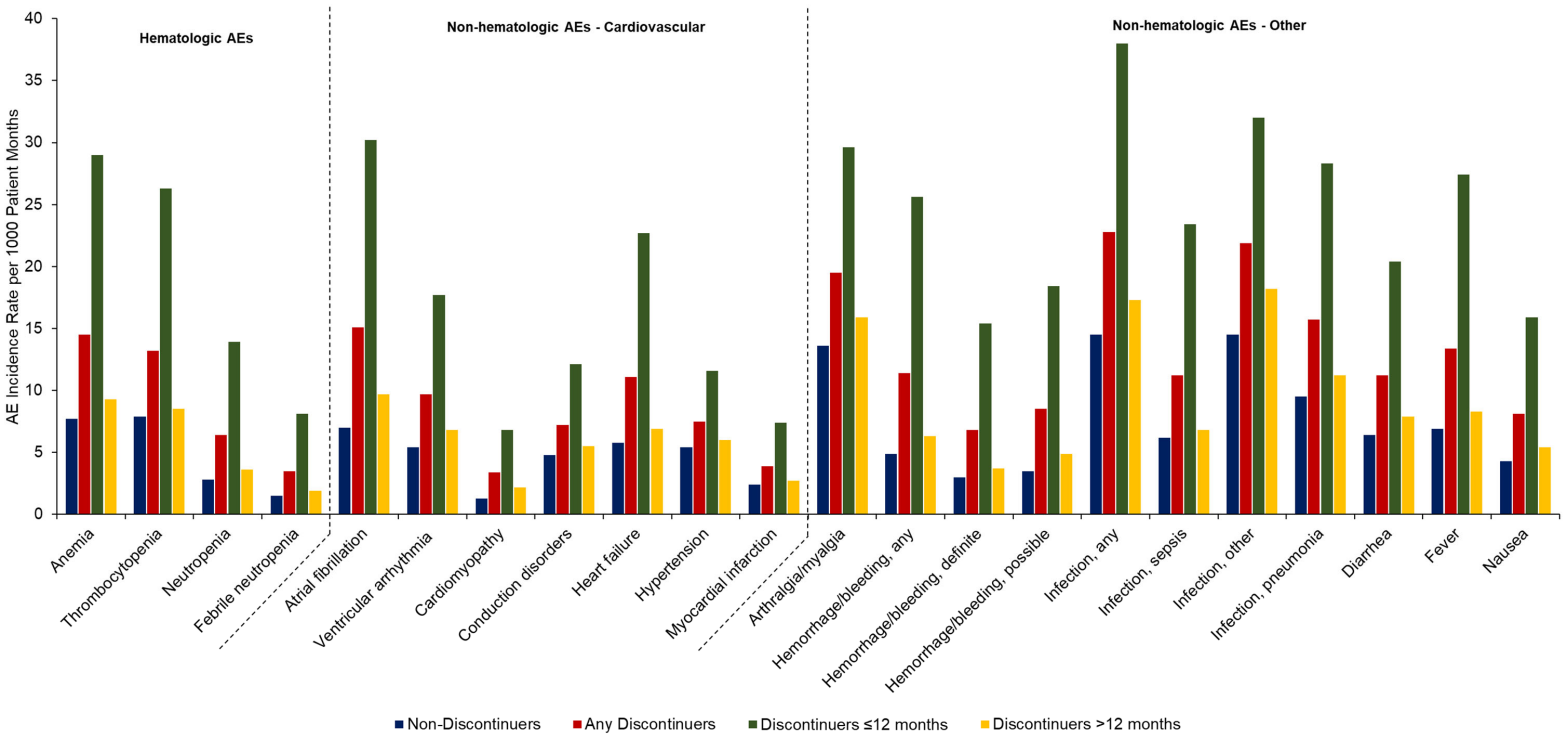
Incidence of individual adverse events per 1000 patient‐months of ibrutinib treatment among elderly Medicare beneficiaries with CLL/SLL initiating ibrutinib by discontinuation status and timing of discontinuation*. The total number of person‐months on ibrutinib treatment for non‐discontinuers (i.e., total patient‐months from ibrutinib initiation date until end of follow‐up) were 107,967. Total number of person‐months on ibrutinib treatment for discontinuers (i.e., total patient‐months from ibrutinib initiation date until its discontinuation) were 85,132 for any discontinuers, 22,551 for discontinuers ≤12 months and 62,581 for discontinuers >12 months. *See Table S3 for data values.

To our knowledge, this is the first real‐world study in a national sample of elderly Medicare patients initiating ibrutinib to demonstrate a substantially higher AE incidence rate among patients who discontinued ibrutinib versus non‐discontinuers, particularly among patients who discontinued within 12 months of ibrutinib initiation. The overall incidence rate including all examined AEs was nearly double for all discontinuers relative to non‐discontinuers and over four times higher for the subgroup of discontinuers ≤12 months relative to non‐discontinuers. Given such a large difference in the incidence rate of AEs, increased clinical attention and mitigation strategies—particularly in the first 12 months after initiation—may help in preventing premature discontinuation of treatment. In particular, we found that cardiovascular comorbidities occurred at a significantly higher rate among discontinuers compared with non‐discontinuers. For instance, the rate of atrial fibrillation was more than four times higher among patients who discontinued within 12 months compared with non‐discontinuers. Our findings support recent calls for improved management of ibrutinib‐related atrial fibrillation among patients with cancer.^11^, ^12^ In addition to increased clinical attention and strategies to mitigate such AEs, treatment considerations in elderly patients with CLL should include substitution of ibrutinib with equally or more effective drugs with lower side effects such as the newer generation BTKis.^13^

It also worth noting that our study results may be an underestimate of the true burden of ibrutinib‐related AEs experienced in elderly Medicare beneficiaries with CLL. A substantial proportion of patients had evidence of CLL‐specific and cardiovascular comorbidities in the 12 months prior to ibrutinib initiation. Given that our AE incident rate only identified new cases of these comorbidities, patients with prior history of these conditions could not be classified as suffering from these ibrutinib‐related AEs. However, it is possible that the use of ibrutinib in these patients worsened pre‐existing comorbidities (e.g., hypertension or atrial fibrillation). Hence, additional efforts are needed to examine the ibrutinib‐related AE experience of patients with such pre‐existing comorbidities and identify best management practices. It would also be worthwhile to examine the characteristics of elderly patients more likely to experience AEs in the real‐world setting who may be suitable for targeted AE management or prevention strategies or are appropriate candidates for other effective drugs with fewer side effects. Finally, while our study was focused on ibrutinib, the first approved and most commonly used BTKi to date, future work should assess whether discontinuation rates have decreased over time as prescriber experience has increased and whether newer generation BTKis have more favorable real‐world AE rates.

As with any administrative database study, claims are subject to possible coding errors and lack detailed clinical information observed in medical charts. Additionally, while our definition of discontinuation (presence of a consecutive 60‐day gap in ibrutinib treatment) has been used in prior analyses,^9^, ^10^ we could not account for phenomena such as dose modification wherein a provider may ask a patient to extend their existing prescription, thus extending the days' supply beyond what would be indicated on the claim. Furthermore, Medicare claims data do not report the reason for discontinuation. We were also unable to assess whether ibrutinib was received in the front‐line or relapsed/refractory setting given a limited 12‐month lookback period for patients in our sample. However, this is offset by the ability to perform efficient and timely population‐level analyses of a nationally representative sample of elderly patients with CLL/SLL compared to studies using data sources with detailed clinical information that typically have smaller, non‐representative samples and may require more resources and time to complete. To our knowledge, our study offers the largest cohort of ibrutinib‐treated patients (*N* = 11,870) to date that is multiple folds higher than the sample sizes in previously published clinical trials or real‐world evidence studies of ibrutinib‐related AEs.^2^, ^3^, ^5^ Some AEs reported in our study (fever, nausea, diarrhea) may be viewed as less severe and providers may not be consistently coding them (and most often under‐reporting them) in the medical claims since they potentially do not impact reimbursement. Hence, our observed incidence rate for these AEs may be underestimated. While our study found substantially higher rates of AE in discontinuers ≤12 months, we are unable to definitively ascertain in claims data whether discontinuation occurred due to AEs or disease progression. Furthermore, our findings from this Medicare study are not generalizable to younger or commercially insured individuals.

In conclusion, our study found that ibrutinib discontinuation was common in older adults with CLL and those with early discontinuation had high incidence of adverse events. Future work should evaluate whether improved management of ibrutinib‐related AEs and/or new treatments can improve real‐world outcomes in elderly patients requiring BTKis for CLL.

## AUTHOR CONTRIBUTIONS

**Scott F. Huntington:** Formal analysis (equal); investigation (equal); methodology (equal); writing – review and editing (equal). **Enrico de Nigris:** Formal analysis (equal); investigation (equal); methodology (equal); writing – review and editing (equal). **Justin T. Puckett:** Formal analysis (equal); investigation (equal); methodology (equal); project administration (lead); writing – original draft (equal). **Sachin Kamal‐Bahl:** Conceptualization (lead); formal analysis (equal); investigation (equal); methodology (equal); writing – original draft (equal). **Mohammed Farooqui:** Formal analysis (equal); investigation (equal); methodology (equal); writing – review and editing (equal). **Katherine Ryland:** Formal analysis (equal); investigation (equal); methodology (equal); writing – review and editing (equal). **Eric M. Sarpong:** Formal analysis (equal); investigation (equal); methodology (equal); writing – review and editing (equal). **Siyang Leng:** Formal analysis (equal); investigation (equal); methodology (equal); writing – review and editing (equal). **Xiaoqin Yang:** Formal analysis (equal); investigation (equal); methodology (equal); writing – review and editing (equal). **Jalpa A. Doshi:** Formal analysis (equal); investigation (equal); methodology (equal); writing – review and editing (equal).

## FUNDING INFORMATION

This study was funded by Merck Sharp & Dohme LLC, a subsidiary of Merck & Co., Inc., Rahway, New Jersey, USA.

## CONFLICT OF INTEREST STATEMENT

SFH: consultancy for Janssen, Pharmacyclics, AbbVie, AstraZeneca, Flatiron Health Inc., Novartis, SeaGen, Genetech, Merck, TG Therapeutics, ADC Therapeutics, Epizyme, Servier, Arvinas, and Thyme Inc.; research funding from Celgene, DTRM Biopharm, and TG Therapeutics; honoraria form Pharmacyclics and AstraZeneca, Bayer; EDN, MF, KR, ES, SL, and XY are employees of Merck Sharp & Dohme LLC, a subsidiary of Merck & Co., Inc., Rahway, New Jersey, USA; JP and SKB are employees of COVIA Health Solutions, a consulting form with clients in the biotech/pharmaceutical industry; JAD: consultancy for AbbVie, Acadia, Janssen, Merck, Otsuka, and Takeda; research funding from Janssen, Merck, and Spark Therapeutics.

## ETHICS STATEMENT

This study was deemed exempt from review by Pearl IRB as it was an analysis of secondary data and posed minimal risk to patients.

## Supporting information


Tables S1–S4.

## Data Availability

This study utilized Medicare claims data obtained from the Centers for Medicare and Medicaid Services and cannot be shared externally per the terms of the data use agreement.

## References

[1] Hallek M . Chronic lymphocytic leukemia: 2017 update on diagnosis, risk stratification, and treatment. Am J Hematol. 2017;92(9):946‐965. doi:10.1002/ajh.24826 28782884

[2] O'Brien SM , Byrd JC , Hillmen P , et al. Outcomes with ibrutinib by line of therapy and post‐ibrutinib discontinuation in patients with chronic lymphocytic leukemia: phase 3 analysis. Am J Hematol. 2019;94(5):554‐562. doi:10.1002/ajh.25436 30767298 PMC6593416

[3] Shanafelt TD , Wang XV , Hanson CA , et al. Long‐term outcomes for ibrutinib‐rituximab and chemoimmunotherapy in CLL: updated results of the E1912 trial. Blood. 2022;140(2):112‐120. doi:10.1182/blood.2021014960 35427411 PMC9283968

[4] Woyach JA , Ruppert AS , Heerema NA , et al. Ibrutinib regimens versus chemoimmunotherapy in older patients with untreated CLL. N Engl J Med. 2018;379(26):2517‐2528. doi:10.1056/NEJMoa1812836 30501481 PMC6325637

[5] Mato AR , Nabhan C , Thompson MC , et al. Toxicities and outcomes of 616 ibrutinib‐treated patients in the United States: a real‐world analysis. Haematologica. 2018;103(5):874‐879. doi:10.3324/haematol.2017.182907 29419429 PMC5927982

[6] Mato AR , Nabhan C , Barr PM , et al. Outcomes of CLL patients treated with sequential kinase inhibitor therapy: a real world experience. Blood. 2016;128(18):2199‐2205. doi:10.1182/blood-2016-05-716977 27601462

[7] Hardy‐Abeloos C , Pinotti R , Gabrilove J . Ibrutinib dose modifications in the management of CLL. J Hematol Oncol. 2020;13(1):66. doi:10.1186/s13045-020-00870-w 32503582 PMC7275592

[8] Sharman J , Kabadi SM , Clark J , Andorsky D . Treatment patterns and outcomes among mantle cell lymphoma patients treated with ibrutinib in the United States: a retrospective electronic medical record database and chart review study. Br J Haematol. 2021;192(4):737‐746. doi:10.1111/bjh.16922 33095453

[9] To TM , Yeh WS , Biondo J , Masaquel AS . Patterns of Ibrutinib use, discontinuation, and hospitalization among patients with chronic lymphocytic leukemia (CLL) in a US healthcare claims database. Blood. 2018;132(Supplement 1):5909. doi:10.1182/blood-2018-99-112679

[10] Hampel PJ , Ding W , Call TG , et al. Rapid disease progression following discontinuation of ibrutinib in patients with chronic lymphocytic leukemia treated in routine clinical practice. Leuk Lymphoma. 2019;60(11):2712‐2719. doi:10.1080/10428194.2019.1602268 31014142 PMC6813846

[11] Ganatra S , Sharma A , Shah S , et al. Ibrutinib‐associated atrial fibrillation. JACC Clin Electrophysiol. 2018;4(12):1491‐1500. doi:10.1016/j.jacep.2018.06.004 30573111

[12] Awan FT , Addison D , Alfraih F , et al. International consensus statement on the management of cardiovascular risk of Bruton's tyrosine kinase inhibitors in CLL. Blood Adv. 2022;6(18):5516‐5525. doi:10.1182/bloodadvances.2022007938 35790105 PMC9631706

[13] Bond DA , Woyach JA . Targeting BTK in CLL: beyond Ibrutinib. Curr Hematol Malig Rep. 2019;14(3):197‐205. doi:10.1007/s11899-019-00512-0 31028669

