# Supplementary material for: Real‐world analysis of adverse event rates after initiation of ibrutinib among Medicare beneficiaries with chronic lymphocytic leukemia
Source: Cancer Med. 2024 Feb 1;13(2):e6953. doi: 10.1002/cam4.6953 (PMC10832339; doi:10.1002/cam4.6953)
Supplement: Supplementary file 1 — Tables S1–S4. [file CAM4-13-e6953-s001.docx]

**Appendix Table 1. List of AEs**

| **Hematologic** | **ICD-9 Codes** | **ICD-10 Codes** | **Source** |
| --- | --- | --- | --- |
| Anemia | 2853, 2850, 2858, 2859 | D641, D642, D643, D6481, D6489, D649 | Denese (2011)^b^; Kilgore (2021)^c^ |
| Thrombocytopenia | 28730, 28731, 28739, 28741, 28749, 2875, 4466 | D692, D693, D6949, D6951, D6959, D696, M311 | Denese (2011)^b^; Kilgore (2021)^c^ |
| Neutropenia^a^ | 28800, 28802, 28803, 28804, 28809 | D701, D702, D703, D704, D708, D709 | Denese (2011)^b^; Kilgore (2021)^c^ |
| **Non-hematologic** |  |  |  |
| *Cardiovascular* |  |  |  |
| Atrial fibrillation | 42731 | I480, I481, I482, I4891, I4811, I4819, I4820, I4821 | Ye (2018)^d^; CMS Chronic Conditions Warehouse Algorithm^e^ |
| Ventricular arrhythmia | 42789, 4279, 4271, 4274, 42741, 42742 | I470, I472, I4901, I4902, I498, I499 | Ye (2018)^d^; CMS ICD-9/ICD-10 Crosswalk^f^ |
| Cardiomyopathy | 4254, 4259 | I425, I427, I429 | Allen (2014)^g^; CMS ICD-9/ICD-10 Crosswalk^f^ |
| Conduction disorders | 426 | I44, I45 | Du (2009)^h^; CMS ICD-9/ICD-10 Crosswalk^f^ |
| Heart failure | 39891, 40201, 40211, 40291, 40401, 40403, 40411, 40413, 40491, 40493, 4280, 4281, 42820, 42821, 42822, 42823, 42830, 42831, 42832, 42833, 42840, 42841, 42842, 42843, 4289 | I0981, I110, I130, I132, I501, I5020, I5021, I5022, I5023, I5030, I5031, I5032, I5033, I5040, I5041, I5042, I5043, I509, I50810, I50811, I50812, I50813, I50814, I5082, I5083, I5084, I5089 | CMS Chronic Conditions Warehouse Algorithm^e^ |
| Hypertension | 36211, 4010, 4011, 4019, 40200, 40210, 40290, 40300, 40301, 40310, 40311, 40390, 40391, 40400, 40402, 40410, 40412, 40490, 40492, 40501, 40509, 40511, 40519, 40591, 40599, 4372 | H35031, H35032, H35033, H35039, I10, I119, I120, I129, I1310, I1311, I150, I151, I152, I158, I159, I674, N262 | CMS Chronic Conditions Warehouse Algorithm^e^ |
| Myocardial Infarction | 41001, 41011, 41021, 41031, 41041, 41051, 41061, 41071, 41081, 41091 | I2101, I2102, I2109, I2111, I2119, I2121, I2129, I213, I214, I220, I221, I222, I228, I229, I219, I21A1, I21A9 | CMS Chronic Conditions Warehouse Algorithm^e^ |
| *Non-cardiovascular* |  |  |  |
| Arthralgia/myalgia | 71950, 71951, 71952, 71953, 71954, 71955, 71956, 71957, 71958, 71959, 72885, 72887, 7291, 72982 | M25511, M25512, M25519, M25521, M25522, M25529, M25531, M25532, M25539, M25551, M25552, M25559, M25561, M25562, M25569, M609, M6281, M62838, M629, M791, M79601, M79602, M79603, M79604, M79605, M79606, M79609, M79621, M79622, M79629, M79631, M79632, M79639, M79641, M79642, M79643, M79644, M79645, M79646, M79651, M79652, M79659, M79661, M79662, M79669, M79671, M79672, M79673, M79674, M79675, M79676, R252 | Schulman (2016)^i^; CMS ICD-9/ICD-10 Crosswalk^f^ |
| Diarrhea | 78791, 5645 | R197, K591 | Buono (2017)^j^; CMS ICD-9/ICD-10 Crosswalk^e^ |
| Hemorrhage/bleeding |  |  |  |
| Hemorrhage/bleeding, possible | 5311, 5313, 5315, 5317, 5319, 5321, 5323, 5325, 5327, 5329, 5331, 5333, 5335, 5337, 5339, 5341, 5343, 5345, 5347, 5349, 53500, 53510, 53520, 53530, 53540, 53550, 53560, 455, 56200, 56201, 56210, 56211, 5301, 2800, 2851, 2859, 79092 | K64, D500, D62, K209, K251, K253, K255, K257, K259, K261, K263, K265, K267, K269, K271, K273, K275, K277, K279, K281, K283, K285, K287, K289, K2900, K2920, K2930, K2940, K2950, K2960, K2980, K5710, K5730, K5732, R791 | Graham (2014)^k^ |
| Hemorrhage/bleeding, definite | 5310, 5312, 5314, 5316, 5320, 5322, 5324, 5326, 5330, 5332, 5334, 5336, 5340, 5342, 5344, 5346, 53501, 53511, 53521, 53531, 53541, 53551, 53561, 53783, 4560, 45620, 5307, 53082, 5780, 4552, 4555, 4558, 56202, 56203, 56212, 56213, 56881, 5693, 56985, 5781, 5789, 59381, 5997, 6238, 6262, 6266, 430, 431, 432, 4320, 4321, 4329, 8520, 8522, 8524, 8530, 4230, 4590, 7191, 7847, 7848, 7863 | I609, I619, I621, I6200, I629, I8501 , R58, K226, K250, K252, K254, K256, K260, K262, K264, K266, K270, K272, K274, K276, K280, K282, K284, K286, K625, K920, K921, K922, R319, N898, N920, N921, M2500, R040, R041, R042, S065, S066, S064, S0636, I8511, K228, K2901, K2921, K2931, K2941, K2951, K2961, K2981, K31811 , K5711, K5713, K5731, K5733, K661, K5521, N280, I609, I619, I621, I6200, I629, I8501 , R58, K226, K250, K252, K254, K256, K260, K262, K264, K266, K270, K272, K274, K276, K280, K282, K284, K286, K625, K920, K921, K922, R319, N898, N920, N921, M2500, R040, R041, R042, S065, S066, S064, S0636, I8511, K228, K2901, K2921, K2931, K2941, K2951, K2961, K2981, K31811 , K5711, K5713, K5731, K5733, K661, K5521, N280 | Graham (2014)^k^ |
| Infection |  |  |  |
| Infection, sepsis | 99591, 99592 | A021, A227, A267, A327, A400, A401, A403, A408, A409, A4101, A4102, A411, A412, A413, A414, A4150, A4151, A4152, A4153, A4159, A4181, A4189, A419, A427, A5486, B377 | Kilgore (2021)^c^ |
| Infection, other | 2, 20, 21, 22, 23, 29, 30, 31, 32, 320, 321, 322, 323, 324, 329, 38, 39, 4, 40, 41, 42, 43, 48, 49, 8, 80, 800, 801, 802, 803, 804, 809, 81, 82, 83, 84, 841, 842, 843, 844, 845, 846, 847, 849, 85, 86, 861, 862, 863, 864, 865, 866, 867, 869, 88, 34, 340, 341, 35, 360, 36, 361, 362, 363, 364, 3640, 3641, 3642, 3643, 368, 3681, 3682, 3689, 390, 391, 392, 393, 394, 398, 399, 400, 401, 402, 403, 404, 4041, 4042, 408, 4081, 4082, 4089, 4112, 4119, 412, 413, 414, 4141, 4142, 4143, 4149, 415, 416, 417, 418, 4181, 4182, 4183, 4184, 4185, 4186, 4189, 419, 101, 1128, 11281, 11282, 11283, 11284, 11285, 114, 1141, 1142, 1143, 1144, 1145, 1149, 115, 11501, 11502, 11503, 11504, 11509, 1151, 11511, 11512, 11513, 11514, 11519, 1159, 11591, 11592, 11593, 11594, 11599, 116, 1160, 1161, 1162, 1170, 1171, 1172, 1173, 1174, 1175, 1176, 1177, 1178, 1179, 118, 3200, 3201, 3202, 3203, 3207, 3208, 32081, 32082, 32089, 3209, 3210, 3211, 3212, 3213, 3214, 3218, 3240, 3241, 3249, 3600, 36000, 36001, 36002, 36003, 36004, 3801, 38010, 38011, 38012, 38013, 38014, 38015, 38016, 3802, 38022, 38023, 3830, 38300, 38301, 38302, 3831, 3832, 38320, 38321, 38322, 376, 3760, 37600, 42099, 421, 4210, 4211, 461, 4610, 4611, 4612, 4613, 4618, 4619, 462, 463, 475, 481, 4820, 4821, 4822, 4823, 48230, 48231, 48232, 48239, 4824, 48240, 48241, 48242, 48249, 4828, 48281, 48282, 48283, 48284, 48289, 485, 486, 49121, 494, 4940, 4941, 510, 5100, 5109, 513, 5130, 5131, 5225, 5227, 5264, 5273, 5283, 540, 5400, 5401, 5409, 541, 542, 56201, 56203, 56211, 56213, 566, 5670, 5671, 5672, 56721, 56722, 56723, 56729, 5673, 56731, 56738, 56739, 5678, 56781, 56782, 56789, 5679, 5750, 5751, 57510, 57511, 57512, 5900, 59000, 59001, 5901, 59010, 59011, 5902, 5903, 5908, 59080, 59081, 5909, 5990, 601, 6010, 6011, 6012, 6751, 67510, 67511, 67512, 67513, 67514, 680, 6800, 6801, 6802, 6803, 6804, 6805, 6806, 6807, 6808, 6809, 681, 6810, 68100, 6811, 68110, 682, 6820, 6821, 6822, 6823, 6824, 6825, 6826, 6827, 6828, 6829, 683, 6850, 686, 6860, 68600, 68601, 68609, 6861, 6868, 6869, 711, 7110, 71100, 71101, 71102, 71103, 71104, 71105, 71106, 71107, 71108, 71109, 72886, 730, 7300, 73000, 73001, 73002, 73003, 73004, 73005, 73006, 73007, 73008, 73009, 7301, 73010, 73011, 73012, 73013, 73014, 73015, 73016, 73017, 73018, 73019, 7302, 73020, 73021, 73022, 73023, 73024, 73025, 73026, 73027, 73028, 73029, 7303, 73030, 73031, 73032, 73033, 73034, 73035, 73036, 73037, 73038, 73039, 7307, 73070, 73071, 73072, 73073, 73074, 73075, 73077, 73078, 73079, 7308, 73080, 73081, 78552, 7854, 7907, 9583, 99591, 99592, 9966, 99660, 99661, 99662, 99663, 99664, 99665, 99666, 99667, 99668, 99669, 9985, 99851, 99859, 9993, 99931, 99932, 99933, 99934, 99939 | A01, A0100, A011, A012, A013, A014, A020, A021, A022, A0220, A0221, A0222, A0223, A0224, A0229, A028, A029, A030, A031, A032, A033, A038, A039, A040, A041, A042, A043, A044, A045, A046, A047, A048, A049, A080, A0811, A0819, A082, A0831, A0832, A0839, A088, A227, A267, A327, A389, A390, A391, A394, A3950, A3951, A3952, A3953, A3981, A3982, A3983, A3989, A40,, A400, A401, A403, A408,, A409, A41, A4152, A419, A420, A421, A422, A427, A4281, A4282, A4289, A429, A438, A439, A46, A480, A481, A483, A4851, A4852, A488, A5486, A690, A691, B375, B376, B377, B3781, B3782, B3784, B380, B381, B382, B383, B384, B387, B388, B389, B393, B394, B395, B399, B409, B410, B419, B420, B421, B427, B429, B439, B449, B450, B451, B457, B459, B469, B470, B471, B479, B480, B481, B482, B488, B49, E832, G02, G060, G061, G062, H0500, H32, H440, H44009, H4401, H44011, H44012, H44013, H44019, H4402,, H44021, H44022, H44023,, H44029, H4409, H6000, H6010, H6020, H60319, H60329, H60339, H60399, H60509, H60519, H60529, H60549, H60559, H60599, H6060, H608X1, H6090, H6193, H6240, H628X1, H70009, H7001,, H70011, H70012, H70013, H70019, H70091, H70092, H70093, H70099, H7010, H70209, H70219, H70229, I308, I32, I330, I39, I96, J0100, J0110, J0120, J0130, J0140, J0190, J020, J029, J0300, J0390, J13, J14, J150, J151, J1520, J15211, J15212, J1529, J153, J154, J155, J156, J158, J180, J181, J189, J36, J441, J471, J479, J850, J851, J852, J853, J860, J869, K046, K047, K122, K352, K353, K3580, K3589, K36, K37, K5712, K5713, K5732, K5733, K610, K611, K613, K650, K651, K652, K653, K654, K658, K659, K67, K6812, K6819, K689, K810, K811, K812, K819, K857, K9081, K9402, K9412, L0202, L0203, L0212, L0213, L02221, L02222, L02223, L02224, L02225, L02226, L02229, L02231, L02232, L02233, L02234, L02235, L02236, L02239, L0233, L02429, L02439, L02529, L02539, L02629, L02639, L02821, L02828, L02831, L02838, L0292, L0293, L03019, L03029, L03039, L03049, L03119, L03129, L03211, L03212, L03221, L03222, L03317, L03319, L03329, L03811, L03818, L03891, L03898, L0390, L0391, L049, L0501, L0502, L080, L081, L0889, L089, L88, L980, M00039, M00049, M00059, M00069, M00079, M0009, M00129, M00139, M00149, M00159, M00169, M00179, M0019, M00229, M00239, M00249, M00259, M00269, M00279, M0029, M00829, M00839, M00849, M00859, M00869, M0087, M00871, M00872, M00879, M0088, M0089, M009, M4620, M60009, M8610, M86119, M86129, M86139, M86149, M86159, M86169, M86179, M8618, M8619, M8620, M86219, M86229, M86239, M86249, M86259, M86269, M8628, M8629, M8660, M86619, M86629, M86639, M86642, M86659, M86669, M86679, M8668, M8669, M869, M8960, M89619, M89629, M89639, M89649, M89659, M89679, M8968, M8969, M9080, M90819, N10, N110, N118, N12, N151, N159, N16, N2884, N2885, N2886, O91111, O91112, O91113, O91119, O9112, R652, R6520, R6521, R7881, T79XXA, T80211A, T80212A, T80219A, T8022XA, T8029XA, T826XXA, T827XXA, T8351XA, T8359XA, T836XXA, T8450XA, T8460XA, T847XXA,, T847XXD, T847XXS, T857, T8571XA, T8579XA, T8579XD, T8579XS, T859, T880XXA | Kilgore (2021)^c^ |
| Infection, pneumonia | 11515, 11595, 1304, 1363, 480, 4801, 4802, 4803, 4808, 4809, 482, 4821, 4822, 4823, 322, 203, 204, 205, 521, 551, 730, 11505, 4800, 481, 4820, 48230, 48231, 48232, 48239, 4824 , 48240, 48241, 48242, 48249, 4828 , 48281, 48282, 48283, 48284, 48289, 4829, 483 , 4830, 4831, 4838, 484 , 4841, 4843, 4845, 4846, 4847, 4848, 485, 486 , 4870, 48801, 48811, 48881, 5163, 51630, 51631, 51632, 51633, 51635, 51636, 51637, 5171 | A003, A0222, A202, A310, A3701, A3711, A3781, A3791, A430, A5484, B012, B052, B0681, B250, B583, B59, B7781, J09X1, J09X9, J100, J1000, J1001, J1008, J1083, J110, J1100, J1108, J12, J120, J121, J122, J123, J128, J1281, J1289, J129, J13, J14, J15, J150, J151, J1520, J1521, J15211, J15212, J1529, J153, J154, J155, J156, J157, J158, J159, J16, J160, J168, J17, J18, J180, J181, J182, J188, J189, J82, J8410, J8411, J84111, J84112, J84114, J84115, J84116, J84117, J8417, J842, J84848, J8489, J849, J851, T6591XA, T6591XD, T6591XS | Kilgore (2021)^c^ |
| Fever | 7806, 78060 , 78062  , 78063, 78066, 78061, 7806 | R502, R5081 , R5082, R5083, R5084, R509 | Kilgore (2021)^c^ |
| Nausea | 78702, 78701, 78703 | R110 , R112, R111, R1110, R1111, R1112, R1113, R1114, R1115 | Kilgore (2021)^c^ |

***Notes:***

*^a^ Febrile neutropenia was a subset of neutropenia who also had evidence of ICD-9 or ICD-10 codes indicative of fever and infection. This approach is consistent with Kilgore et al. (2021).*

*^b^ Denese MD et al. An observational study of outcomes after initial infused therapy in Medicare patients diagnosed with chronic lymphocytic leukemia. Blood. 2011 Mar 31; 117(13): 3505–3513.*

*^c^ Kilgore KM et al. Burden of illness and outcomes in second-line large B-cell lymphoma treatment: real-world analysis of Medicare beneficiaries. Future Oncol. 2021 Dec;17(35):4837-4847.*

*^d^ Ye Y et al. Algorithms used to identify ventricular arrhythmias and sudden cardiac death in retrospective studies: a systematic literature review. Ther Adv Cardiovasc Dis. 2018 Feb;12(2):39-51.*

*^e^ CMS Chronic Conditions Warehouse. Chronic Condition Categories.* [*https://www2.ccwdata.org/web/guest/condition-categories-chronic*](https://www2.ccwdata.org/web/guest/condition-categories-chronic)

*^f^ NBER. ICD-9 CM to and from ICD-10-CM Crosswalk for General Equivalence Mappings.* [*https://www.nber.org/research/data/icd-9-cm-and-icd-10-cm-and-icd-10-pcs-crosswalk-or-general-equivalence-mappings*](https://www.nber.org/research/data/icd-9-cm-and-icd-10-cm-and-icd-10-pcs-crosswalk-or-general-equivalence-mappings)

*^g^ Allen LA et al. Performance of claims-based algorithms for identifying heart failure and cardiomyopathy among patients diagnosed with breast cancer. Med Care. 2014 May;52(5):e30-8.*

*^h^ Du XL et al. Cardiac toxicity associated with anthracycline-containing chemotherapy in older women with breast cancer. Cancer. 2009 Nov 15;115(22):5296-308.*

*^i^ Schulman KL et al. Development and Validation of Algorithms to Identify Statin Intolerance in a US Administrative Database.* *Value Health. 2016 Sep-Oct;19(6):852-860.*

*^j^ Buono JL et al. Economic Burden of Irritable Bowel Syndrome with Diarrhea: Retrospective Analysis of a U.S. Commercially Insured Population. J Manag Care Spec Pharm. 2017 Apr;23(4):453-460.*

*^k^ Graham DJ et al. Cardiovascular, bleeding, and mortality risks in elderly Medicare patients treated with dabigatran or warfarin for nonvalvular atrial fibrillation. Circulation. 2015 Jan 13;131(2):157-64.*

**Appendix Table 2. Sample Attrition Table**

| **Criteria** | **N** | **%** |
| --- | --- | --- |
| ≥1 claim for ibrutinib filled in a standalone Part D plan between **January 1, 2014 and December 31, 2018**. The first ibrutinib prescription during this time frame will be designated the **index date**. | 25,658 |  |
| Continuous fee-for-service Medicare Part A and B coverage for at least 12 months before the index date | 20,838 | 81.2% |
| Continuous fee-for-service Medicare Part D coverage for at least 12 months before the index date | 19,489 | 93.5% |
| Continuous fee-for-service Medicare Part A and B coverage for at least 4 months or until death after the index date | 19,194 | 98.5% |
| Continuous fee-for-service Medicare Part D coverage for at least 4 months or until death after the index date | 19,170 | 99.9% |
| ≥1 claim with a diagnosis of CLL/SLL in the primary diagnosis position on or before the index date (i.e., pre-index period) and ≥1 claim with a diagnosis of CLL/SLL in the post-index period | 13,602 | 71.0% |
| ≥66 years old on index date | 12,792 | 94.0% |
| **NO** Evidence of ≥1 claim with a diagnosis for another FDA-approved indication for ibrutinib in the primary diagnosis position on the index date or in the 12-months before the index date (i.e., pre-index period) and ≥1 claim with a diagnosis for another FDA-approved indication for ibrutinib in the primary diagnosis position in the post-index follow-up period. Approved indications for ibrutinib will include mantle cell lymphoma [MCL], marginal zone lymphoma [MZL], Waldenström macroglobulinemia, and chronic graft versus host disease. | 11,941 | 93.3% |
| No evidence of ≥1 claim for ibrutinib in the 12 months prior to the index date (i.e., to ensure “new” initiators of ibrutinib on the index date) | 11,870 | 99.4% |
| **Study Groups Based on Discontinuation Status:** |  |  |
| Non-discontinuers | 4,128 | 34.8% |
| Any discontinuers | 7,742 | 65.2% |
| **Study Groups Based on Timing of Discontinuation:** |  |  |
| Non-discontinuers | 4,128 | 34.8% |
| Discontinuers ≤12 months | 5,356 | 45.1% |
| Discontinuers >12 months | 2,386 | 20.1% |

**Appendix Table 3. Sample Characteristics by Discontinuation Status and Timing of Discontinuation Among Elderly Medicare Beneficiaries with CLL/SLL Initiating Ibrutinib**

| **Characteristic** | **Non-Discontinuers** | **Discontinuers** | | |
| --- | --- | --- | --- | --- |
|  |  | **Any** | **≤12 months** | **>12 months** |
| **N** | 4128 | 7742 | 5356 | 2386 |
| Length of available follow-up in years from ibrutinib initiation date |  |  |  |  |
| Median (IQR) | 1.9 (1.1, 3.1) | 2.2 (1.2, 3.4) | 1.7 (0.9, 2.8) | 3.2 (2.3, 4.3) |
| Mean (SD) | 2.1 (1.4) | 2.4 (1.5) | 2.0 (1.4) | 3.3 (1.3) |
| Age, mean (SD) | 76.4 (6.7) | 77.6 (6.9) | 78.1 (7.0) | 76.3 (6.5) |
| <75 years | 1836 (44.5%) | 2878 (37.2%) | 1837 (34.3%) | 1041 (43.6%) |
| ≥75 years | 2292 (55.5%) | 4864 (62.8%) | 3519 (65.7%) | 1345 (56.4%) |
| Male | 2474 (59.9%) | 4470 (57.7%) | 3090 (57.7%) | 1380 (57.8%) |
| Race |  |  |  |  |
| White | 3698 (89.6%) | 7002 (90.4%) | 4881 (91.1%) | 2121 (88.9%) |
| Black | 282 (6.8%) | 492 (6.4%) | 304 (5.7%) | 188 (7.9%) |
| Other | 148 (3.6%) | 248 (3.2%) | 171 (3.2%) | 77 (3.2%) |
| Census Region |  |  |  |  |
| Northeast | 937 (22.7%) | 1621 (20.9%) | 1134 (21.2%) | 487 (20.4%) |
| Midwest | 1052 (25.5%) | 1883 (24.3%) | 1291 (24.1%) | 592 (24.8%) |
| South | 1445 (35.0%) | 2849 (36.8%) | 1956 (36.5%) | 893 (37.4%) |
| West | 694 (16.8%) | 1389 (17.9%) | 975 (18.2%) | 414 (17.4%) |
| Ibrutinib received as monotherapy | 3726 (90.3%) | 6761 (87.3%) | 4612 (86.1%) | 2149 (90.1%) |
| Ibrutinib dose at initiation ≥420 mg | 3763 (91.2%) | 7049 (91.0%) | 4817 (89.9%) | 2232 (93.5%) |
| Evidence of prior CLL treatment in 12-month pre-index period | 1136 (27.5%) | 2783 (35.9%) | 1917 (35.8%) | 866 (36.3%) |
| Type of prior CLL treatments in 12-month pre-index period* |  |  |  |  |
| Cytotoxic chemotherapy | 648 (15.7%) | 1687 (21.8%) | 1131 (21.1%) | 556 (23.3%) |
| Anti-CD20s | 943 (22.8%) | 2252 (29.1%) | 1558 (29.1%) | 694 (29.1%) |
| PI3Ks | 20 (0.5%) | 67 (0.9%) | 49 (0.9%) | 18 (0.8%) |
| NCI-adjusted Charlson comorbidity score, mean (SD) | 1.3 (1.6) | 1.5 (1.8) | 1.7 (1.8) | 1.2 (1.6) |
| Comorbidities in pre-index period |  |  |  |  |
| CLL-specific comorbidities |  |  |  |  |
| Anemia | 1854 (44.9%) | 3716 (48.0%) | 2655 (49.6%) | 1061 (44.5%) |
| Thrombocytopenia | 1405 (34.0%) | 2877 (37.2%) | 2024 (37.8%) | 853 (35.8%) |
| Neutropenia | 525 (12.7%) | 1155 (14.9%) | 798 (14.9%) | 357 (15.0%) |
| Febrile neutropenia | 172 (4.2%) | 302 (3.9%) | 219 (4.1%) | 83 (3.5%) |
| Cardiovascular comorbidities |  |  |  |  |
| Atrial fibrillation | 574 (13.9%) | 1486 (19.2%) | 1164 (21.7%) | 322 (13.5%) |
| Ventricular arrhythmia | 440 (10.7%) | 889 (11.5%) | 645 (12.0%) | 244 (10.2%) |
| Cardiomyopathy | 144 (3.5%) | 360 (4.6%) | 274 (5.1%) | 86 (3.6%) |
| Conduction disorders | 328 (7.9%) | 802 (10.4%) | 607 (11.3%) | 195 (8.2%) |
| Heart failure | 584 (14.1%) | 1393 (18.0%) | 1099 (20.5%) | 294 (12.3%) |
| Hypertension | 2966 (71.9%) | 5800 (74.9%) | 4123 (77.0%) | 1677 (70.3%) |
| Myocardial infarction | 72 (1.7%) | 212 (2.7%) | 169 (3.2%) | 43 (1.8%) |
| All-cause hospitalization in pre-index period | 1403 (34.0%) | 2954 (38.2%) | 2171 (40.5%) | 783 (32.8%) |
| * Other types of CLL treatment were also evaluated such as BCL-2, but these results cannot be reported due to CMS rules prohibiting the reporting of cell sizes <11. | | | | |

**Appendix Table 4. Rate of Adverse Events per 1000 Patient-Months of Ibrutinib Treatment Among Elderly Medicare Beneficiaries with CLL/SLL Initiating Ibrutinib by Discontinuation Status**

| **Characteristic** | **Non-Discontinuers** | **Discontinuers** | | |
| --- | --- | --- | --- | --- |
|  |  | **Any** | **≤12 months** | **>12 months** |
| **Overall Sample** |  |  |  |  |
| Number of patients | 4128 | 7742 | 5356 | 2386 |
| Number of **patient-months** from ibrutinib initiation date until discontinuation for discontinuers or until end of follow-up for non-discontinuers | 107,967 | 85,132 | 22,551 | 62,581 |
| **Incidence rate of adverse events per 1000 patient-months*** |  |  |  |  |
| Type of adverse event |  |  |  |  |
| Hematologic |  |  |  |  |
| Anemia | 7.7 | 14.5 | 29.0 | 9.3 |
| Thrombocytopenia | 7.9 | 13.2 | 26.3 | 8.5 |
| Neutropenia | 2.8 | 6.4 | 13.9 | 3.6 |
| Febrile neutropenia | 1.5 | 3.5 | 8.1 | 1.9 |
| Non-hematologic |  |  |  |  |
| Cardiovascular |  |  |  |  |
| Atrial fibrillation | 7.0 | 15.1 | 30.2 | 9.7 |
| Ventricular arrhythmia | 5.4 | 9.7 | 17.7 | 6.8 |
| Cardiomyopathy | 1.3 | 3.4 | 6.8 | 2.2 |
| Conduction disorders | 4.8 | 7.2 | 12.1 | 5.5 |
| Heart failure | 5.8 | 11.1 | 22.7 | 6.9 |
| Hypertension | 5.4 | 7.5 | 11.6 | 6.0 |
| Myocardial infarction | 2.4 | 3.9 | 7.4 | 2.7 |
| Non-cardiovascular |  |  |  |  |
| Arthralgia/myalgia | 13.6 | 19.5 | 29.6 | 15.9 |
| Hemorrhage/bleeding, any | 4.9 | 11.4 | 25.6 | 6.3 |
| Hemorrhage/bleeding, definite | 3.0 | 6.8 | 15.4 | 3.7 |
| Hemorrhage/bleeding, possible | 3.5 | 8.5 | 18.4 | 4.9 |
| Infection, any | 14.5 | 22.8 | 38.0 | 17.3 |
| Infection, sepsis | 6.2 | 11.2 | 23.4 | 6.8 |
| Infection, other | 14.5 | 21.9 | 32.0 | 18.2 |
| Infection, pneumonia | 9.5 | 15.7 | 28.3 | 11.2 |
| Diarrhea | 6.4 | 11.2 | 20.4 | 7.9 |
| Fever | 6.9 | 13.4 | 27.4 | 8.3 |
| Nausea | 4.3 | 8.1 | 15.9 | 5.4 |
| Any of the above adverse events of interest | 32.9 | 62.5 | 140.2 | 34.5 |
